# Randomized clinical trial to evaluate the pathogenicity of *Bibersteinia trehalosi* in respiratory disease among calves

**DOI:** 10.1186/1746-6148-10-89

**Published:** 2014-04-18

**Authors:** Christy J Hanthorn, Reneé D Dewell, Vickie L Cooper, Timothy S Frana, Paul J Plummer, Chong Wang, Grant A Dewell

**Affiliations:** 1 Department of Veterinary and Diagnostic Production Animal Medicine, College of Veterinary Medicine, Iowa State University, 2237 Lloyd Vet Med, Ames, IA 50011, USA; 2 Center for Food Security and Public Health, Department of Veterinary Microbiology and Preventive Medicine, College of Veterinary Medicine, Iowa State University, Ames, IA 50011, USA; 3 Department of Veterinary Microbiology and Preventive Medicine, College of Veterinary Medicine, Iowa State University, Ames, IA 50011, USA; 4 Department of Statistics, College of Liberal Arts and Sciences, Iowa State University, Ames, IA 50011, USA

**Keywords:** *Bibersteinia trehalosi*, Bovine respiratory disease, Calf, Pneumonia, Pasteurellosis

## Abstract

**Background:**

*Bibersteinia trehalosi* causes respiratory disease in ruminants particularly in wild and domestic sheep. Recently, there has been an increased number of *B. trehalosi* isolates obtained from diagnostic samples from bovine respiratory disease cases. This study evaluated the role of *B. trehalosi* in bovine respiratory disease using an intra-tracheal inoculation model in calves. Thirty six cross bred 2–3 month old dairy calves were inoculated intra-tracheally with either leukotoxin negative *B. trehalosi*, leukotoxin positive *B. trehalosi* isolate, *Mannheimia haemolytica*, a combination of leukotoxin negative *B. trehalosi* and *M. haemolytica* or negative control. Calves were euthanized and necropsy performed on day 10 of study.

**Results:**

*B. trehalosi* inoculated calves did not have increased lung involvement compared to control calves. Additionally, *B. trehalosi* was only cultured once from the lungs of inoculated calves at necropsy.

**Conclusions:**

Based on these findings *B. trehalosi* may not be a primary pathogen of respiratory disease in cattle. Culture of *B. trehalosi* from diagnostic submissions should not be immediately identified as a primary cause of respiratory disease.

## Background

*Bibersteinia trehalosi* is a known pathogen of ruminants and has been identified worldwide [1]. *B. trehalosi* was formerly included in a single species *of Pasteurella haemolytica* as biotype T [2]. This pathogen was recently reclassified as *B. trehalosi* on the basis of phylogenetic studies [3]. *B. trehalosi* has been associated with systemic pasteurellosis or septicaemia in lambs [4] and pneumonia in bighorn sheep (Ovis canadensis) [5,6].

Although *B. trehalosi* infections are considered rare in cattle, the agent is occasionally isolated from bovine lungs. Diagnostic reports of severe non-responsive Bovine Respiratory Disease (BRD) outbreaks associated with multi-drug resistant *B. trehalosi* have been documented [7]. Clinical manifestations of these strains are often associated with multi-drug resistance and severe lung pathology. The reasons for development of these potentially highly virulent strains remain unclear. It has been hypothesized that *B. trehalosi* may have acquired increased pathogenicity from other bacteria. Some reports in the literature suggest that *B. trehalosi* and *Mannheimia haemolytica* can share genetic material [8,9]. Earlier work has shown that nucleotide diversity of lktA from *B. trehalosi* is minimal (0.7%) compared to *M. haemolytica* (22.0%) and were genetically different from M*. haemolytica*[10]. However, this report included only *B. trehalosi* isolates from ovine samples. Bovine adapted isolates of *B. trehalosi* may have acquired some genetic material from *M. heamolytica* increasing its ability to infect cattle.

Understanding the role different pathogens play in BRD is critical to understanding and effectively treating clinical cases. The primary objective of this study was to evaluate the pathogenicity of *B. trehalosi* in respiratory disease among calves using field strains of *B. trehalosi*.

## Methods

Two *B. trehalosi* isolates were identified from diagnostic submissions of bovine cases for the challenge study. Isolate identification was confirmed by 16S ribosomal RNA analysis. One isolate was PCR positive for the leukotoxin (lktA) gene [11] and the other isolate was negative. The lktA gene was amplified with a 5′ primer lktA9 (5′-TCAAGAAGAGCTGGCAAC-3′) and the 3′ primer lktA7 (5′-AGTGAGGGCAACTAAACC-3′). Amplification parameters were: denaturation at 94°C for 45 s, annealing at 62°C for 45 s, and extension at 72 C for 2 min. The leukotoxin-PCR-positive *B. trehalosi* isolate was cultured from the lungs, along with a *Pasteurella multocida,* from a feedlot calf that had been treated with antibiotics multiple times for Bovine Respiratory Disease that had fibrinosuppurative bronchopneumonia from a group of 273 kg Southeastern feedlot calves with 16% respiratory morbidity and 10% mortality. The leukotoxin-PCR-negative *B. trehalosi* isolate was cultured from the brain of a bull with multifocal, suppurative meningoencephalitis.

Thirty six 8–12 week old individually housed Holstein cross steer calves were obtained from a private calf raiser. Calves were tested for persistent infection by BVDV by immunohistochemistry prior to purchase and confirmed PI negative. Nasal swab samples were collected from each calf and submitted for bacterial culture to the diagnostic lab prior (Day −1) to inoculation. During the experimental challenge, calves were housed in a Biosecurity Level 2 facility at the ISU Livestock Infectious Disease Isolation Facility. Each calf was confined separately in raised 0.9 × 1.8 meter pens that provided no opportunity for calf-to-calf contact in a room that held 12 calves at a time. Three groups of calves were utilized to accommodate room layout. Calves were provided free choice water and were fed mixed grass hay and a pre-mixed calf starter. Biosecurity procedures such as changing protective gloves and clothing between calves were employed by all research personnel working with calves. Calves were randomly assigned by a random number generator [12] to one of five treatments. Treatments were randomly assigned to 1 of 3 replicates (Table 1). Eight calves were assigned to each bacterial challenge treatment and 4 calves to a negative control treatment.

**Table 1:** Replicate and treatment group assignments

	**Replicate 1**	**Replicate 2**	**Replicate 3**
Control Calves	1	1	2
Leukotoxin negative *B. trehalosi*	3	3	2
Leukotoxin postive *B. trehalosi*	3	3	2
*M. haemolytica*	3	2	3
*M. haemolytica* and leukotoxin negative *B. trehalosi*	2	3	3

Assignment of calves to treatment groups by replicate of study presented in table format.

The 5 treatment groups were:

1) Negative control (Brain Heart Infusion broth),

2) Leukotoxin negative *B. trehalosi*,

3) Leukotoxin positive *B. trehalosi*,

4) *M. haemolytica*

5) Combination of leukotoxin negative *B. trehalosi* and *M. haemolytica*

Prior to inoculation, each isolate was grown overnight on two blood agar plates and then transferred to a Brain Heart Infusion broth immediately prior to inoculation to obtain an estimated 2.5 × 10^9^ CFU of bacteria per ml. Isolates for group 5 had an estimated 2.5 × 10^9^ CFU of bacteria per ml for both leukotoxin negative *B. trehalosi* and *M. haemolytica* for a total of 5 × 10^9^ CFU of bacteria per ml. A small sample of the inoculum was enumerated on blood agar plates to determine exact concentration of the inoculum. Inoculums ranged on average from 1.7 × 10^9^ to 3.3 × 10^9^ CFU of bacteria per ml of broth. A sterile 90 cm number 8 French Foley catheter (MILA International) was used to inoculate each calf. The researcher administering the inoculum was masked to the treatment being administered. For each inoculation, a sterile catheter was passed intranasal to approximately the bifurcation of the trachea and 20 ml of the inoculum was infused. Following inoculation, the calf’s head was briefly elevated.

Following inoculation, calves were monitored twice daily by trained personnel masked to treatment on Days 0–9. Measurements recorded included rectal temperature, pulse rate and respiration rate. In addition each calf was clinically assessed and assigned a respiratory and depression score during twice daily monitoring. The following scoring systems were used for the study.

Respiratory scores:

0 – Normal, eyes are clear, nose is clean with no discharge, normal breathing

1 – Mild Respiratory, serous discharge from eyes and\or nose, slight cough

2 – Moderate Respiratory, muco-purulent discharge, cough, increased respiratory rate

3 – Severe Respiratory, excessive muco-purulent discharge, harsh cough, open mouth breathing

Depression scores

0 – Normal, cattle are bright and alert, hold their head up and readily move away from the observer

1 – Mild depression, cattle’s attitude is slightly depressed but respond quickly to observer and appear normal

2 – Moderate depression, cattle stand with head down, ears droop, abdomen lack of fill and may appear floppy, cattle move away slowly from observer

3 – Severe depression, cattle stand with head down and very reluctant to move, very noticeable gauntness of abdomen

Any calf that was assigned a respiratory and/or depression score of three was considered a candidate for euthanasia. If calf was determined to be moribund from physical exam, it was humanly euthanized. All surviving calves were euthanized on day 9 of the study by use of captive bolt followed by pithing. All euthanasias followed American Veterinary Medical Association guidelines [13].

Immediately following euthanasia, a necropsy was performed on each calf. The respiratory system was evaluated by a diagnostic pathologist. The percent abnormal lung was visually estimated for each lobe and total lung involvement calculated as a percentage of total lung as described by Jericho and Langford [14]. Samples of representative lung as determined by the pathologist were submitted ISU Veterinary Diagnostic Laboratory for routine culture and histology. For culture, samples were plated on bovine blood agar and incubated aerobically at 35°C, sheep blood agar and incubated anaerobically at 35°C and tergitol-7 agar and incubated aerobically at 35°C. All samples were incubated overnight and suspect hemolytic and non-hemolytic colonies were selected for identification by matrix-assisted laser desorption/ionization--time of flight (MALDI-TOF).

This protocol was approved by the Iowa State University Institutional Animal Care and Use Committee (#5-12-7352-B) and the Institutional Biosafety Committee (#12-I-0017-A). Personnel conducting daily monitoring, the pathologist and the statistician were blinded to calves treatment group.

### Statistical analysis

Our goal was to describe the magnitude and variation of our collected measurements and assess the association of these variables with BRD and *B. trehalosi.* The primary outcome of interest included percent lung involvement, temperature, depression score and respiratory score. The percent abnormal lung response was analyzed using mixed effect Analysis of Variance (ANOVA) model, with Treatment as fixed effect and Group as a random effect. The remaining responses (Temperature, Depression score, and Respiratory score) were analyzed using repeated measures ANOVA model, with Treatment, time and their interaction as fixed effects and group as random effect. Comparisons among treatment groups were performed using Tukey’s t-tests. SAS® Version 9.2 (SAS® Institute Inc., Cary, NC, USA) was used in analyses. A p-value <0.05 was considered significant.

## Results

Thirty five of the thirty six candidates were enrolled in the study. One calf assigned to the *M. haemolytica* group died prior to enrollment in study. *Pasturella multocida* was isolated from nasal swab samples from 34 of 35 calves. *M. haemolytica* was isolated from 8 of 35 calves. *M. haemolytica* positive nasal swab calves were fairly evenly spread across all five treatment groups (1/4 in Control group, 1/8 in Leukotoxin negative *B. trehalosi* group*,* 2/8 Leukotoxin positive *B. trehalosi* group*,* 1/7 *M. haemolytica* group, 3/8 leukotoxin negative *B. trehalosi* and *M. haemolytica* combination group). Six of the 35 enrolled calves were euthanized prior to day 9 of the study. Three of the euthanized calves (3/7) in *M. haemolytica* group (days 1, 2 and 2) and three calves (3/8) from the leukotoxin negative *B. trehalosi* and *M. haemolytica* combination treatment (days 2, 4 and 7) were euthanized according to protocol because of a clinical assessment of moribund. Overall, the mean estimated percent lung involvement was highest for the *M. haemolytica* group (49%) followed by the mixed infection of *M. haemolytica* and leukotoxin negative *B. trehalosi* (26%). The leukotoxin postive *B. trehalosi* had a mean 18% estimated lung involvement while the leukotoxin negative *B. trehalosi* mean lung involvement was estimated to be 13%. The control group had a mean lung involvement estimate of 13% (Table 2). Calves with lung involvement had histologic lesions that were consistent with bronchointerstitial pneumonia (26 of 35 calves) and 20 of these calves also had evidence of pyogranulomatus pneumonia.

**Table 2:** Percent lung involvement by treatment group

	**Control**	**Leukotoxin negative *B. trehalosi***	**Leukotoxin postive *B. trehalosi***	***M. haemolytica***	***M. haemolytica *** **and leukotoxin negative *B. trehalosi***
Calves	20.4	0	36.55	57.25	71.6
	26.2	24.25	27.1	53.95	61.3
	0	21.7	28.1	26	62.85
	4.4	32.1	27.1	33.15	0
		18.05	14.2	28.6	1.8
		6.15	1.5	63.6	0.6
		3.6	11.35	75.1	4.75
		0	1.45		6.1
Mean*	12.75 a	13.2 a	18.4 a	48.2 b	26.1 a, b
Adj P value**		0.9999	0.9981	0.0715	0.8049

*means with different letters are statistically different (p < 0.05).

**Adj p value of treatment from the control group.

Individual and mean lung involved in disease process as a percentage of total lung volume. Treatment group means that were statistically different from other treatment groups are indicated by different letters (a or b) in table. Adjusted p-values of treatment group from control group is reported.

*M. haemolytica* was isolated from5 of 7 lungs of the *M. haemolytica* inoculated group and 4 of 8 lungs from the leukotoxin negative *B. trehalosi* and *M. haemolytica* combination group. *B. trehalosi* was isolated from one of 24 lungs that were inoculated with *B. trehalosi*. Calf number 6 in the leukotoxin negative *B. trehalosi* and *M. haemolytica* combination inoculation treatment group was euthanized on day 2 of the study as reported above. The calf had 71.6% lung involvement and both *M. haemolytica* and a *B. trehalosi* were isolated from the lung tissue. *B. trehalosi* was not isolated from the lung tissue of any other calves that were inoculated with a *B. trehalosi* isolate.

**Figure 1:**
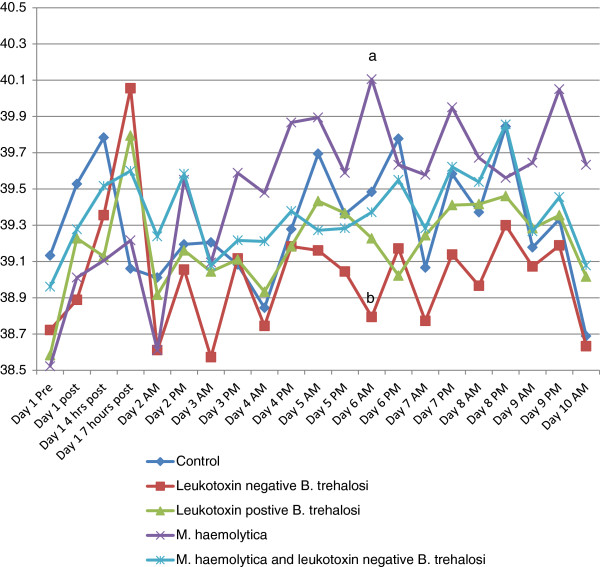
**Temperature over time by treatment group.** Line graph of rectal temperature by treatment group for each time point when temperature was recorded. Time points that were statistically different are indicated by different letters (a or b).

**Figure 2:**
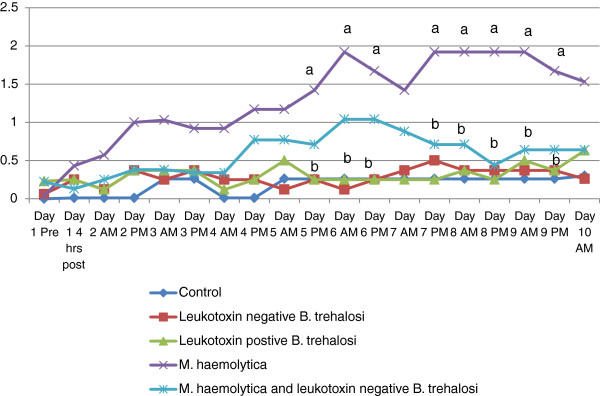
**Depression score over time by Treatment group.** Line graph of depression score by treatment group for each time point when depression score was recorded. Time points that were statistically different are indicated by different letters (a or b).

**Figure 3:**
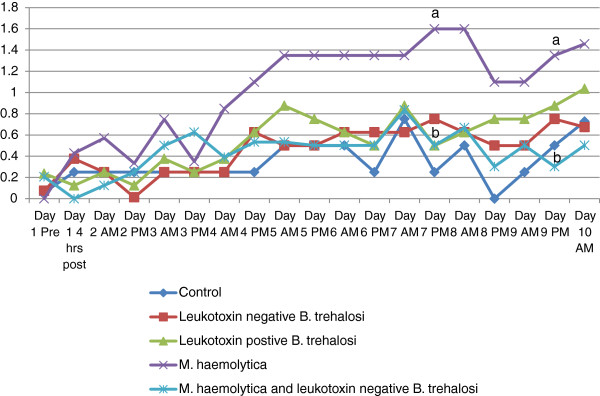
**Respiratory score over time by treatment group.** Line graph of respiratory score by treatment group for each time point when respiratory score was recorded. Time points that were statistically different are indicated by different letters (a or b).

Rectal temperature (Figure 1), depression scores (Figure 2) and respiratory scores (Figure 3) followed an expected pattern after inoculation. Overall, rectal temperatures ranged from 35.3-41.3°C. Depression scores ranged from 0–3 and respiratory scores ranged from 0–3.There were occasional time periods when differences in rectal temperatures, depression score or respiratory score between the *M. haemolytica* and the *B. trehalosi* or control groups were statistically significant (Figures 1, 2 and 3).

The calves from both groups inoculated with *B. trehalosi* demonstrated increased rectal temperature (>39.5°C) at 7 hours after inoculation compared to calves that had been inoculated with *M. haemolytica*. Temperatures for the two *B. trehalosi* only treatment groups returned to normal levels (37.5 – 39.5°C) within 12 hours. Calves in the *M. haemolytica* only inoculation group did not demonstrate increased rectal temperatures (>39.5°C) on average until 24 hours following inoculation and temperatures of these calves remained elevated except for morning of day 2. Calves in the combined inoculation group fluctuated above and below 39.5°C throughout the study (Figure 1).

Depression scores (Figure 2) for the *M. haemolytica* inoculation group began to increase 4 hours post inoculation and remained elevated at 1 or greater throughout the study. The combined inoculation group did not have increased depression scores (≥1) until Day 3 of the study and never reached the severity of the *M. haemolytica* only inoculation group. Neither of the *B. trehalosi* inoculation groups had elevated depression scores. The average depression score for the control group was 0.2. Both *B. trehalosi* only inoculation groups had an average depression score of 0.3 while the *M. haemolytica* inoculation group had an average of 1.3. The combined inoculation group had an average depression score of 0.6.Respiratory scores for the *M. haemolytica* inoculation group ranged from 0–3 and were not consistently abnormal (≥1) until Day 3 of the study. None of the other groups had elevated average respiratory scores (Table 3).

**Table 3:** Average depression and respiratory score by treatment group

	**Average peak depression score (Range)**	**Average peak respiratory score (Range)**
Control	0.25 (0–1)	1 (1–1)
Leukotoxin negative *B. trehalosi*	0.75 (0–2)	1 (0–1)
Leukotoxin postive *B. trehalosi*	1 (0–2)	1.25 (1–2)
*M. haemolytica*	2 (0–3)	1.7 (1–3)
*M. haemolytica* and leukotoxin negative *B. trehalosi*	1.25 (0–3)	1.25 (0–3)

Average depression and respiratory scores over the course of the study are reported for each treatment group in table format.

## Discussion

Results of this study indicate that *B. trehalosi* isolates used in this study are not associated with significant disease in a research setting. Calves inoculated with either a leukotoxin positive (PCR positive for the leukotoxin (lktA) gene ) or leukotoxin negative isolate of *B. trehalosi* did not demonstrate increased lung involvement compared to control calves. This finding contradicts some perceptions that *B. trehalosi* is an emerging primary respiratory pathogen of cattle [7]. In contrast, this study’s results are more supportive of *B. trehalosi’s* role in BRD as secondary and perhaps opportunistic bacteria. Reports of *B. trehalosi* associated pneumonia are from diagnostic submissions, often without access to history of inciting causes of respiratory disease, nutritional health of calves, additional stressors or information on antimicrobial therapies. In this challenge model healthy calves were challenged with field isolates of *B. trehalosi* and extensive lung involvement was not identified post inoculation.

The limitations of this study include the small size of the study, isolate credibility, the inoculation method and the health status of the calves prior to inoculation. The study was designed as a pilot study to evaluate the pathogenicity of *B. trehalosi* in cattle. The number of calves in each group was limited as much as possible to decrease the number of calves sacrificed while still providing important information. The small size of the study decreases the statistical power to identify statically significant differences even though numerically the difference between the negative control group (inoculated with broth only) and the positive control group (*M. haemolytica*) were substantial.

The authors are unaware of any *B. trehalosi* isolates that have demonstrated pathogenicity in a challenge model in cattle. The *B. trehalosi* isolates selected for this study originated from field submissions to a diagnostic laboratory. Our approach was to use leukotoxin positive and negative *B. trehalosi* isolates that had been associated with respiratory disease and could potentially be associated with lung pathology in the study calves.

Though unlikely, it is possible that, during the inoculation procedure, the dose was inadvertently misplaced into the esophagus instead of the lungs. There are multiple bovine respiratory disease challenge models cited in the literature including trans-tracheal injection, trans-thoracic injection, bronchoscope and using a nasal-tracheal approach that this study utilized. Confirmation that the catheter was correctly placed in the trachea as opposed to the esophagus can be difficult. However, the *M. haemolytica* positive control group, had substantial lung involvement and a p-value of 0.07 compared to the negative control group suggesting that the inoculation technique and the challenge model was valid.

Calves in the study were conventionally reared dairy crossbred calves. Calves were purchased from a calf raiser and were considered clinically normal at the time of purchase and did not exhibit signs of respiratory disease at enrollment. However, two calves in the control group had lung lesions (20–26% lung involvement). Thus, this pathology was presumed to have resulted from a previous infection not associated with inoculation. Evidence of lung pathology from prior respiratory disease in some control calves may confound results between treatment groups. Histologic lesions characteristic of a previous BRSV infection was seen in 17 of the calves. Since two control calves demonstrated some lung pathology, the potential difference between treatment groups and the control group was affected.

Pre-study pharyngeal swabs did not identify any calves carrying *B. trehalosi* and the calf farm did not have a history of *B. trehalosi* disease. Despite this, it is still possible that calves could have had exposure to *B. trehalosi* prior to enrollment in the project. Given the age of the calves, it is also possible that passive immunity played a role in immune response. However, there is currently not a validated serologic test for cattle to determine previous exposure to *B. trehalosi*. Therefore, it is possible that calves may have had previous unknown exposure to *B. trehalosi* that enhanced their immunological response.

It was surprising that calves inoculated with a combination of *M. haemolytica* and *B. trehalosi* tended to have numerically less lung involvement than that of *M. haemolytica* inoculation alone (difference was not significant). One would expect that mixed infections would potentiate increased lung involvement and subsequent pathology. Other research has suggested that *B. trehalosi* can inhibit the growth of *M. haemolytica*. Dassanayake et al. [15] reported that *B. trehalosi* inhibited the growth of *M. haemolytica* in co-culture when *B. trehalosi* enters the stationary phase. One possible explanation for this finding is that; when both bacteria are inoculated simultaneously, *B. trehalosi* may inhibit *in vivo* growth of *M. haemolytica* until the immune system removes *B. trehalosi*. This postulated in vivo inhibition may delay the onset of clinical signs of BRD (such as a temperature spike and decrease the subsequent severity of lung involvement.

Our goal was to determine if *B. trehalosi* is really an emerging primary BRD pathogen as has been reported or if infection occurs secondary to some other viral/bacterial insult. Cortese et al. [7] have reported peracute fatal pneumonia in healthy adult cattle attributed to *B. trehalosi*. Other, anecdotal reports concerning *B. trehalosi* and BRD, suggest that *B. trehalosi* is associated with severe fibinous pneumonia with consolidation (especially caudal lobes) and pleuritis that is unresponsive to antimicrobial therapy. The more minor role that *B. trehalosi* may play in BRD is supported by the isolation results described in this study. *B. trehalosi* was isolated from only one set of lungs post inoculation and in that case the sample was obtained only 3 days following inoculation from a calf that had been inoculated with both *B. trehalosi* and *M. haemolytica*. This finding suggests that *B. trehalosi* may not persist long term in bovine lung. The temperature response of calves inoculated with *B. trehalosi* or *M. haemolytica* was different (p < 0.05) over time. The early spike and rapid return to normal in rectal temperature from the *B. trehalosi* inoculated calves may indicate that the immune system in the lungs may more rapidly identify and respond to a *B. trehalosi* bacterial insult compared to the *M. haemolytica* inoculated calves. The authors acknowledge that *B. trehalosi* isolates have been obtained from samples submitted from field cases. These “positives” suggest the occurrence of an opportunistic infection and proliferation of *B. trehalosi* after normal lung defenses have been compromised by other primary respiratory pathogens. The plausibility of *B. trehalosi* as a secondary, perhaps even an opportunistic, pathogen is a stronger argument than *B. trehalosi* as an emerging primary BRD pathogen of economic importance.

## Conclusion

This pilot study suggests that *B. trehalosi* may not be an important primary pathogen of respiratory disease in cattle. The study’s results suggest that *B. trehalosi* is likely a secondary, or perhaps opportunistic, BRD pathogen. Culture of this bacterium from diagnostic submissions should not necessarily be interpreted cited as a primary cause of respiratory disease but may still have a role as a secondary pathogen or an opportunistic invader. Clinicians and diagnosticians should temper a *B. trehalosi* culture in context of the clinical setting. Practically, identification of *B. trehalosi* as the primary isolate from a submitted lung sample may suggest a failure in identification or treatment of a major BRD pathogen such as M. haemolytica. Further challenge studies designed to assess *B.trehalosi’s* interaction with other BRD pathogens (IBR, BVDV, *Mycoplasma bovis*, *Histophilus somni*) should be conducted. Additionally, a challenge model designed to evaluate if *B. trehalosi*, inoculated after *M. haemolytica* has already initiated lung damage, can lead to more severe BRD would be beneficial.

## Competing interests

The authors declare that they have no competing interests.

## Authors’ contributions

GD and PP conceived and coordinated the experiment. CH and RD performed the experiment. VC evaluated lungs. CW conducted statistical analysis. TF helped with bacteriology guidance and draft of the manuscript. All authors read and approved the final manuscript.
